# The landscape of exosomal non-coding RNAs in breast cancer drug resistance, focusing on underlying molecular mechanisms

**DOI:** 10.3389/fphar.2023.1152672

**Published:** 2023-04-19

**Authors:** Malihe Rezaee, Fatemeh Mohammadi, Atoosa Keshavarzmotamed, Sheida Yahyazadeh, Omid Vakili, Yaser Eshaghi Milasi, Vida Veisi, Rohollah Mousavi Dehmordi, Sepideh Asadi, Seyedeh Sara Ghorbanhosseini, Mehdi Rostami, Mina Alimohammadi, Abbas Azadi, Nushin Moussavi, Zatollah Asemi, Azadeh Aminianfar, Hamed Mirzaei, Alireza Mafi

**Affiliations:** ^1^ Department of Pharmacology, School of Medicine, Shahid Beheshti University of Medical Sciences, Tehran, Iran; ^2^ Tehran Heart Center, Cardiovascular Diseases Research Institute, Tehran University of Medical Sciences, Tehran, Iran; ^3^ Afzalipour Faculty of Medicine, Kerman University of Medical Sciences, Kerman, Iran; ^4^ Student Research Committee, Guilan University of Medical Sciences, Rasht, Iran; ^5^ Department of Immunology, School of Medicine, Shiraz University of Medical Sciences, Shiraz, Iran; ^6^ Autophagy Research Center, Department of Clinical Biochemistry, School of Medicine, Shiraz University of Medical Sciences, Shiraz, Iran; ^7^ Department of Clinical Biochemistry, School of Pharmacy and Pharmaceutical Sciences, Isfahan University of Medical Sciences, Isfahan, Iran; ^8^ School of Medicine, Shahrekord University of Medical Sciences, Shahrekord, Iran; ^9^ Department of Clinical Biochemistry, Faculty of Medicine, Ahvaz Jundishapur University of Medical Sciences, Ahvaz, Iran; ^10^ Department of Life Science Engineering, Faculty of New Sciences and Technologies, University of Tehran, Tehran, Iran; ^11^ Department of Clinical Biochemistry, Mashhad University of Medical Sciences, Mashhad, Iran; ^12^ Student Research Committee, Department of Immunology, School of Medicine, Shahid Beheshti University of Medical Sciences, Tehran, Iran; ^13^ Department of Internal Medicine, Lorestan University of Medical Sciences, Khorramabad, Iran; ^14^ Department of Surgery, Kashan University of Medical Sciences, Kashan, Iran; ^15^ Research Center for Biochemistry and Nutrition in Metabolic Diseases, Kashan University of Medical Sciences, Kashan, Iran; ^16^ Nutrition and Food Security Research Center, Isfahan University of Medical Sciences, Isfahan, Iran

**Keywords:** breast neoplasms, drug resistance, exosomes, microRNAs, circular RNA, long non-coding RNA

## Abstract

Breast cancer (BC) is the most common malignancy among women worldwide. Like many other cancers, BC therapy is challenging and sometimes frustrating. In spite of the various therapeutic modalities applied to treat the cancer, drug resistance, also known as, chemoresistance, is very common in almost all BCs. Undesirably, a breast tumor might be resistant to different curative approaches (e.g., chemo- and immunotherapy) at the same period of time. Exosomes, as double membrane-bound extracellular vesicles 1) secreted from different cell species, can considerably transfer cell products and components through the bloodstream. In this context, non-coding RNAs (ncRNAs), including miRNAs, long ncRNAs (lncRNAs), and circular RNAs (circRNAs), are a chief group of exosomal constituents with amazing abilities to regulate the underlying pathogenic mechanisms of BC, such as cell proliferation, angiogenesis, invasion, metastasis, migration, and particularly drug resistance. Thereby, exosomal ncRNAs can be considered potential mediators of BC progression and drug resistance. Moreover, as the corresponding exosomal ncRNAs circulate in the bloodstream and are found in different body fluids, they can serve as foremost prognostic/diagnostic biomarkers. The current study aims to comprehensively review the most recent findings on BC-related molecular mechanisms and signaling pathways affected by exosomal miRNAs, lncRNAs, and circRNAs, with a focus on drug resistance. Also, the potential of the same exosomal ncRNAs in the diagnosis and prognosis of BC will be discussed in detail.

## 1 Introduction

Breast cancer (BC), the most common malignancy among women, is the second leading cause of cancer death for women worldwide (DeSantis et al., 2017). The National Breast Cancer Coalition (NBCC) has estimated the incidence of BC to be 12.9% in 2022, and unfortunately, a woman dies from BC every 13 min (Oza et al., 2021). BC survival rates are considerably different among the nations, as high-income countries have reported a 5-year survival of 80%, while low-income nations have reported a 5-year survival of less than 40% (Coleman et al., 2008). Age, family history of BC, reproductive and environmental parameters, and genetic predisposition are considered substantial risk factors for BC onset and progression (Shah et al., 2014). Almost 40% of recurrent BCs are detected in patients without particular symptoms in regular examinations, highlighting the significance of BC management and follow-up. Clinical assessments, such as physical examination, should be carried out every 4–6 months for at least 5 years, and afterward, for every 12 months, along with annual mammography evaluation (Shah et al., 2014; Rautalin et al., 2022).

**TABLE 1:** Exosomal ncRNAs (miRNAs, lncRNAs, and circRNAs) involved in BC drug resistance.

NcRNA	Type of EVs	EV source	Chemotherapy drug	Expression pattern	Corresponding targets/signaling pathway	Clinical application	Reference
MiRNAs							
miR-221/222	Exosome	TAM-resistant MCF-7 cells	Tamoxifen	Up	Direct inhibition of the expression of P27 and ERα	Therapeutic target	Wei et al. (2014)
Several miRNAs, including miR-1246, miR-23a- miR-1469, miR-638, miR-1915, miR-2861, let-7a, let-7b, miR-27a, and miR-16	Exosome	DOC-resistant MCF-7 cells	Docetaxel	Up	Axon guidance, MAPK signaling pathway, Wnt signaling pathway, cell cycle, and TGF-β signaling pathway	Therapeutic target Monitoring biomarker	Chen et al. (2014a)
miR-222	Exosome	DOC-resistant MCF-7 cells	Docetaxel	Up	Underexpression of PTEN	Therapeutic target Monitoring biomarker	Chen et al. (2014b)
miR-134	EV	TNBC aggressive clonal variant (Hs578 Ts(i)_8_) cells	Anti-Hsp90 drugs	Down	Underexpression of STAT5B, Hsp90, and Bcl-2	Therapeutic target Monitoring biomarker Prognostic biomarker	O'Brien et al. (2015)
miR-222/223	Exosome	MSCs	Carboplatin	Up	Cell cycle	Therapeutic target	Bliss et al. (2016)
miR-222	Exosome	ADM-resistant MCF-7 cells	Adriamycin	Up	N/A	Therapeutic target	Yu et al. (2016)
miR-1246	Exosome	Metastatic breast cancer MDA-MB-231 cell	Docetaxel Epirubicin Gemcitabine	Up	Underexpression of CCNG2	Therapeutic target	Li et al. (2017a)
miR-770	Exosome	TNBC cells	Doxorubicin	Down	Underexpression of STMN1	Prognostic biomarker Therapeutic target	Li et al. (2018b)
miR-155	Exosome	CSCs	Doxorubicin Paclitaxel	Up	EMT molecular changes: 1) upregulation of BMI1, SLUG, SNAIL, SOX9, and EZH2	Therapeutic target	Santos et al. (2018)
		DOX-resistant cells			2) repression of E-cadherin		
		PTX-resistant cells (both MCF-7 and MDA-MB-231)			3) downregulation of C/EBP-β, TGF-β, and FOXO-3a		
miR-567	Exosome	MCF-10A (drug-sensitive cells)	Trastuzumab	Down	Atg5 inhibition	Prognostic biomarker Therapeutic target	Han et al. (2020)
miR-1246 and miR-155	Exosome	TZB-resistant BC patients	Trastuzumab	Up	N/A	Monitoring biomarker Prognostic biomarker	Zhang et al. (2020)
miR-1236	Exosome	Adipose MSC	Cisplatin	Down	Suppression of SLC9A1 and the Wnt/β-catenin signaling pathway	Therapeutic target	Jia et al. (2020)
miR-22	Exosome	Cancer-associated fibroblast (CAF)	Tamoxifen	Up	Underexpression of ERα and PTEN	Therapeutic target	Gao et al. (2020)
					Activation of the PI3K/AKT pathway		
miR-9-5p	Exosome	TAM-resistant MCF-7 cells	Tamoxifen	Up	Underexpression of ADIPOQ	Therapeutic target	Liu et al. (2021)
miR-342-3p	Exosome	MSCs	Doxorubicin	Down	Underexpression of ID4 that regulates the EMT process	Therapeutic target	Yu et al. (2022)
			Fluorouracil				
			Cisplatin				
miR-887-3p	EV	Resistant MDA-MB-231 BC cells	Doxorubicin	Up	Underexpression of BTBD7 and activation of the Notch1/Hes1 signaling pathway	Therapeutic target	Wang et al. (2022a)
			Cisplatin				
			Fulvestrant				
miR-423-5p	Exosome	Cisplatin-resistant MDA-MB-231 BC cells	Cisplatin	Up	Overexpression of P-gp and migration and invasion capabilities Inhibition of apoptosis	Therapeutic target	Wang et al. (2019a)
LncRNAs							
LncRNA UCA1	Exosome	TAM-resistant LCC2 cells	Tamoxifen	Up	Inhibition of caspase-3 and apoptosis	Therapeutic target	Xu et al. (2016)
LncRNA APAP2-AS1	Exosome	TZB-resistant BC cells	Trastuzumab	Up	Inhibition of TZB-induced apoptosis	Therapeutic target	Zheng et al. (2019)
HIF-1α-stabilizing long non-coding RNA (HISLA)	EV	Tumor-associated macrophages (TAMs)	Chemotherapy	Up	Stabilization of HIF-1α *via* blocking the interaction between PHD2 and HIF-1α Promotion of aerobic glycolysis and apoptotic resistance	Therapeutic target Chemotherapeutic resistance biomarker Prognostic biomarker	Chen et al. (2019c)
LncRNA H19	Exosome	Doxorubicin-resistant MCF‐7 BC cells	Doxorubicin	Up	Decreasing cell viability	Therapeutic target	Wang et al. (2020)
					Lowering colony‐forming ability	Chemotherapeutic resistance biomarker	
					Inducing apoptosis		
LncRNA AGAP2-AS1	Exosome	TZB-resistant SKBR-3 BC cells	Trastuzumab	Up	Overexpression of Atg10 and autophagy	Therapeutic target Prognostic value	Qian et al. (2021)
CircRNAs							
Circ_UBE2D2	Exosome	TAM-resistant MCF-7 cell line	Tamoxifen	Up	Sponging the miR-200a-3p	Therapeutic target Chemotherapeutic resistance biomarker	Hu et al. (2020b)
Circ-MMP11	Exosome	LAP-resistant BC cells	Lapatinib	Up	Sponging the miR-153-3p to activate ANLN	Therapeutic target	Wu et al. (2021)

Abbreviations: ERα, estrogen receptor alpha; Hsp90, heat shock protein 90; Atg, autophagy-related gene; N/A, not available.

Based upon immunohistochemical staining for key proteins’ expression, breast tumors are considered to contain at least 1% of the following genes: human epidermal growth factor receptor 2 (HER2, *Erbb2* gene), estrogen receptor (ER), and progesterone receptor (PR, *Pgr gene*) (Hammond et al., 2010). Tumors that do not express the three mentioned proteins are referred to as “basal-like” or “triple-negative” breast cancer (TNBC) (Klinge, 2018). Primary breast tumors have predominantly been reported to be ER+/PR+/HER2-, which determine the basis of applying therapeutics, including radiation therapy, surgery, and endocrine therapies (e.g., anti-estrogen therapy) (Klinge, 2018). Nevertheless, 30%–40% of BC cases have been found to be resistant to endocrine therapies and develop metastatic conditions (Ring and Dowsett, 2004; Piggott et al., 2018). According to the PAM50 test that analyzes 50 genes expressed in primary breast tumors, more individualized therapeutics are planned to be used in clinical settings (Cejalvo et al., 2018).

Non-coding RNAs (ncRNAs), which comprise 99% of the total cellular RNAs in human cells (Consortium, 2012; Romano et al., 2017), have recently attracted much attention in relation to BC pathogenesis and development (Sobhani et al., 2022). In brief, ncRNAs are a large family of RNA molecules, classified into two subclasses based on their size: minor or short ncRNAs with less than 200 nucleotides in size (Krichevsky et al., 2003) and major or long ncRNAs with more than 200 nucleotides in size. Multiple ncRNAs are categorized in the aforementioned groups, in which miRNAs, lncRNAs, and circRNAs have been reported to be more essential in cancer pathophysiology (Cech and Steitz, 2014; Beermann et al., 2016). NcRNA molecules are involved in several biological processes, as well as protein coding/decoding, transcription regulation, and gene expression modulation, in both physiological and pathological conditions (Krichevsky et al., 2003; Gregory and Shiekhattar, 2005; Esquela-Kerscher and Slack, 2006; Huarte and Rinn, 2010; Kasinski and Slack, 2011; Rinn and Huarte, 2011; Tay et al., 2014; Anastasiadou et al., 2018). More interestingly, ncRNAs can be packaged into EVs, especially exosomes (Meldolesi, 2018), to be locally or systemically transmitted among the cells (Palazzo and Lee, 2015; Sun et al., 2018a); a characteristic that enables ncRNA transfer from tumor cells to normal cells, and *vice versa*. The specific structure of EVs (i.e., their bilayer membranes) supports the process of ncRNA transmission and protects them against circulatory nucleases and other possible threat factors (Teixeira et al., 2015; Mateescu et al., 2017).

Exosomal ncRNAs have previously been reported to be prominent in BC-related pathogenic mechanisms, such as cell proliferation, invasion, metastasis, migration, and especially drug resistance (Bullock et al., 2015; Xie et al., 2019). In drug resistance, exosomal ncRNAs interfere with various mechanisms, from drug absorption to its efflux. In addition, they can link resistant cells to sensitive ones through their exosome-dependent transmission (Ashekyan et al., 2022). In the current review, the crosstalk between exosomal ncRNAs, including exosomal miRNAs, lncRNAs, and circRNAs, and BC drug resistance is discussed in detail, and the possible roles of the corresponding RNAs in the deceleration or exacerbation of chemoresistance are comprehensively highlighted. Since exosomal ncRNAs are considered circulating agents with diagnostic and therapeutic potential, and regarding the limitations and disadvantages of the current diagnostic/therapeutic strategies, prognostic/diagnostic potentials of the aforestated RNA molecules will also be reviewed in brief.

## 2 BC drug resistance in brief: Underlying mechanisms

During the relapse abundance among BC patients, it seems necessary to elucidate the resistance-related mechanisms in detail (Harbeck and Gnant, 2017; Ji et al., 2019). There are two types of drug resistance; intrinsic resistance and acquired resistance. In intrinsic drug resistance, cancer cannot naturally be targeted by a specific agent, which can be the result of genetic mutations, tumor heterogeneity, or the absence of drug target expression. In the acquired type, therapeutic effectiveness is attenuated over time (Cosentino et al., 2021). Modifications of HER receptor signaling have been reported to have a substantial role in developing BC drug resistance. Lee *et al.* discovered that concentrations of heat shock protein 90 (HSP90) were highly elevated in drug-resistant BC cells, and a mixture of lapatinib and HSP90 inhibitors represented an advantageous therapeutic strategy for HER2+ patients (Lee et al., 2020). As a xenobiotic transporter, the BC-resistant protein (BCRP, also known as, ATP-binding cassette G2 (ABCG2)) contributes to multidrug resistance (MDR) and is responsible for the efflux of anticancer drugs (Szczygieł et al., 2022). In parenthesis, MDR1 is one of the most well-known ABC transporters that induces chemoresistance (Robey et al., 2018). Furthermore, BC heterogeneity in the tumor microenvironment can impact the response to the therapeutic approach, and thus tumor progression (Kim and Zhang, 2016). The increased glucose uptake and disrupted oxidative phosphorylation and glycolysis are linked to cancer progression toward advanced stages, as well as the development of drug resistance against approved chemotherapy drugs, such as paclitaxel, cisplatin, doxorubicin, and tamoxifen (Varghese et al., 2020). Intercellular communications between tumor cells and the surrounding cells, including immune cells, adipocytes, and fibroblasts, significantly affect the increase in resistance against anticancer drugs (Fontana et al., 2022). On the other hand, the enhanced levels of mitogen-activated protein kinase (MAPK), phosphoinositide 3-kinase (PI3K), EGFR, and phospho-ribosomal protein S6 kinase beta-1 (p-S6K1) are linked to BC radioresistance (Gray et al., 2019). Multiple evaluations have shown that a large number of miRNAs, as well as lncRNAs and circRNAs, contribute to the progression of BC drug resistance. However, tumor suppressor ncRNAs, which are silenced in chemoresistant BC, can suppress drug resistance if upregulated (Zhu et al., 2011; Gao et al., 2016).

## 3 Exosomes: Biogenesis and biological features

Exosomes, as small EVs (30–150 nm), are produced during endosomal maturation (Chahar and Casola, 2015). Typically, exosomes range from 1.13 g/mL (derived from B cells) to 1.19 g/m (derived from epithelial cells) in density (Zakharova and Fomina, 2007; Bobrie et al., 2011). Exosomal characteristics, as well as the type of cargos transferred by these EVs, closely depend on the cell of origin and the state involved in exosome generation. Exosomes can carry multiple cargoes, including different RNA molecules, DNA, peptides, and several proteins such as oncoproteins, transcriptional regulators, tumor suppressors, and splicing factors (Valadi and BossiosSjostrandLeeLotvall, 2007). The corresponding EVs facilitate intercellular communications by transferring the aforementioned biologically active molecules (Chahar and Casola, 2015). Structurally, exosomes are produced by endosomes through inward budding from the limited multivesicular body (MVB) membrane (Minciacchi and Di Vizio, 2015). Throughout the process, invagination of the endosomal membrane results in the formation of intraluminal vesicles (ILVs) in large MVBs (Huotari and Helenius, 2011). Through fusion, most ILVs will be released into the extracellular space in the form of exosomes (Yellon and Davidson, 2014). The biogenesis of exosomes involves a set of consecutive molecular machinery, of which the endosomal sorting complex, required for the ESCRT transport machinery, is the most substantial system, playing a crucial role in ILV formation (JH, 2008). ESCRT has four complexes, namely, ESCRT-0, ESCRT-I, ESCRT-II, and ESCRT-III, and the associated proteins, i.e., vacuolar protein sorting-associated protein 4A (VPS4A), tumor susceptibility gene 101 protein (TSG101), and ALG-2-interacting protein X (ALIX), which increase the generation rate of MVBs, vesicle budding (Henne and Emr, 2011), sorting, binding, and clustering of ubiquitinylated proteins and receptors in the late endosomes (Kalra and Mathivanan, 2016). The ESCRT-0 ubiquitin-binding subunits induce the sequestration and recognition of ubiquitinated cargo proteins into the endosomal membrane domains (Henne and Emr, 2011). Facilitating ILV budding, where cargo is transferred into the lumen, can be induced by ESCRT-I and ESCRT-II. ALIX recruits ESCRT-III for the acceleration of pulling, spiral generation, and full budding (Kalra and Mathivanan, 2016). Moreover, VPS4A and TSG101 have regulatory roles in exosome biogenesis through the ESCRT-dependent pathway (Baietti et al., 2012). Eventually, following ILV formation, the ESCRT-III complex is separated from the MVB membrane, while the sorting protein VPS4A supplies its energy (Henne and Emr, 2011). Despite the modulatory roles of ESCRT-associated mechanisms being controversial in exosome release, various ESCRT components, as well as the ubiquitinated proteins, are found in exosomes that are separated from multiple cells (Zhang et al., 2019). Recent studies have also clarified the possible role of the ESCRT-independent pathway in sorting exosomal cargos into MVBs and subsequent biogenesis of exosomes containing lipids and associated proteins, like tetraspanin (Babst, 2011; Stuffers et al., 2019). Unlike ESCRT-mediated protein sorting, it has been shown that RNA loading into exosomes is correlated with cargo domains and self-organizing lipids (Janas et al., 2015). Tetraspanins, as transmembrane proteins (e.g., CD9, CD63, and CD81) found in exosomes, are significantly involved in the ESCRT-independent pathway (Chairoungdua et al., 2010; van Niel and Raposo, 2018). Mechanistically, tetraspanins trigger the organization of membranous microdomains, called tetraspanin-enriched microdomains (TEMs), using several cytosolic and transmembrane signaling proteins (ME, 2003). Recently, both ESCRT-dependent and -independent mechanisms have been reported to cooperate to regulate exosome biogenesis (Maas and Weaver, 2017), which shows the corresponding pathways can work synergistically. The presence of different subpopulations of exosomes may be explained by the presence of different biogenic machineries, as well as different cell species and cellular homeostasis (Zhang et al., 2019). The small size and unified appearance of exosomes help them escape from mononuclear clearance, resulting in their prolonged circulation time to affect cell-to-cell interactions, more efficiently (Zhang et al., 2019). Regarding the role of exosomes as essential mediators of intercellular communications, they have been demonstrated to impact the pathogenesis of several disorders, such as cancers (Colombo and Thery, 2014). Meanwhile, the possible clinical applications of exosomes, especially as diagnostic biomarkers and therapeutic delivery vehicles, have attracted much attention recently (Jiang et al., 2019).

## 4 Non-coding RNAs: A summary of their structure and subclassifications

Coding RNAs (i.e., mRNAs) are known for their ability to encode proteins that can serve as enzymes, signal transductors, transcription factors, etc., while ncRNAs predominantly have regulatory effects (Li and Liu, 2019). However, about 98% of all transcriptional output is ncRNA, and only 2% is responsible for the formation of coding RNAs (Legnini et al., 2017). Although the term ncRNA refers to an RNA molecule without coding capacity, recent evaluations have demonstrated that a number of ncRNAs can surprisingly be translated into proteins (Legnini et al., 2017). NcRNAs are categorized into two major subclasses, according to the nucleotide length, long ncRNAs and small ncRNAs, which are classified into further subgroups (Costa, 2005). Small ncRNAs mostly contribute to post-transcriptional gene regulation, whereas long ncRNAs have roles in epigenetic modifications (Sana et al., 2012). MiRNAs, small nuclear RNAs (snoRNAs); small interfering RNAs (siRNAs); rRNAs; tRNAs; and Piwi-interacting RNAs (piRNAs) are key RNA molecules belonging to the small ncRNA subclass; on the other hand, pseudogenes; antisense RNAs (asRNAs); long intergenic ncRNAs (lincRNAs); and circRNAs belong to the long ncRNA subclass (Chan, 2018). Several investigations have shown that ncRNAs are essential molecules with the ability to affect a wide spectrum of cellular processes, including inflammation, oxidative stress, autophagy, fibrosis, and pathophysiological processes associated with malignancies such as cell proliferation, migration, angiogenesis, and especially drug resistance (Chan, 2018; Li et al., 2019). Moreover, ncRNAs have been found to serve as hallmarks of cancer cells, suggesting their possible role as prognostic and diagnostic biomarkers (Sana et al., 2012). Among multiple ncRNA molecules, miRNAs, lncRNAs, and circRNAs have been identified to be central to the regulation of cancer-related processes, such as drug resistance.

MiRNAs are small single-stranded RNA molecules (20–24 nucleotides) primarily contributing to post-transcriptional gene modulation through binding to the target gene’s 3′-untranslated region (3′UTR) to suppress the translation (Ruan and Ouyang, 2009; Sana et al., 2012; Bahmyari et al., 2021; Mafi et al., 2022). Subcellular localization of miRNAs, the affinity of miRNA-related interconnections, and the number of target mRNAs and miRNAs influence the miRNA–target gene interactions (O'Brien et al., 2018). Extracellular miRNAs, which can be transferred to target cells by vesicles (e.g., exosomes) or through binding to proteins, are key messengers in modulating cell-to-cell communications (Wang and Sen, 2016). Since miRNAs can affect multiple aspects of tumorigenesis such as angiogenesis, immune deregulation, metastasis, and drug resistance, research studies have recently introduced miRNAs as significant biologically active components of exosomes, affecting neoplastic conditions (Hayes and Lawler, 2014; Kozomara and Griffiths-Jones, 2018). Being inside the exosomes protects miRNAs, as well as the other ncRNAs, from RNase-dependent degradation (Lima et al., 2009).

LncRNAs, which are more than 200 nucleotides in length, are not translated into functional proteins but rarely encode small functional peptides. LncRNAs, first observed in eukaryotic cells, are located in the cytoplasm or nucleus (Dempsey and Cui, 2017). According to the chromosomal position, lncRNAs are categorized into aslncRNAs, divergent lncRNAs, enhancer RNAs (eRNAs), intronic lncRNAs, promoter-associated lncRNAs, intergenic lncRNAs, and transcription start site-associated lncRNAs (Hombach and Kretz, 2016). Abnormal expression of lncRNAs has been reported to accelerate cancer progression by interfering with tumorigenesis, cell survival and proliferation, metastasis and invasion, and drug resistance (Hung et al., 2014; Prensner et al., 2014). By being packaged inside the exosomes, lncRNAs can release into the tumor microenvironment and transferred to recipient cells, moderating the process of cancer metastasis and progression (Fan et al., 2018). Exosomal lncRNAs are also considered potential tumor markers based on their specificity and sensitivity (Yousefi et al., 2020).

Eventually, circRNAs are single-stranded covalently closed molecules originating from pre-mRNA back-splicing (Sanger et al., 1976; Jeck et al., 2013; Salami et al., 2022). CircRNAs lack 5’ to 3’ polarity or poly A tail, and thus are less sensitive to RNA exonuclease- or RNase R-related degradation (Jeck and Sharpless, 2014; Chen and Yang, 2015; Najafi et al., 2022); a prominent characteristic making circRNAs potential prognostic and diagnostic biomarkers and therapeutic agents (Zhou et al., 2020). CircRNAs can interfere with pathogenic processes of multiple diseases, including cancer (Li et al., 2020a), neurological disorders (Mehta and Vemuganti, 2020), cardiovascular diseases (ufiero et al., 2019), and autoimmune defects (Zhou et al., 2019). Nevertheless, the underlying mechanisms by which circRNAs affect physiological/pathological circumstances are not fully understood (Zhou et al., 2020). Some circRNAs can act as protein decoys, recruiters, and scaffolds (Xiao and Wilusz, 2020). In addition, circRNAs exert biological functions, like serving as miRNA sponges, transcriptional regulators, and protein templates (Kristensen et al., 2019; Yu and Kuo, 2019; Huang et al., 2020). Beyond the significance of circRNAs, alone, exosomal circRNAs are considerably involved in cancer-associated mechanisms, including cell proliferation, migration, invasion, metastasis, and drug resistance (Wang et al., 2018a).

## 5 Functional roles of exosomal ncRNAs in multiple diseases

As mentioned previously, exosomal ncRNAs can potentially upregulate or downregulate the processes that impact tumor expansion, like cell proliferation, tumor metastasis, invasion, immunomodulation, angiogenesis, and specifically drug resistance (Fan et al., 2018). Also, tumor-suppressed ncRNAs can be transferred by exosomes to other tumor or non-tumor cells (Li and Xu, 2019). In this context, exosomal ncRNAs have been shown to be correlated with various human cancers, such as BC, lung cancer, hepatocellular carcinoma (HCC), glioblastomas, and prostate cancer. (Li et al., 2018a; He et al., 2018; Hu et al., 2020a; Movahedpour et al., 2021; Movahedpour et al., 2022a; Taghvimi et al., 2022). For instance, in the case of HCC, exosomal ncRNAs interfere with the process of liver fibrosis and subsequent cirrhosis and consequently provoke tumorigenesis and HCC development (Quail and Joyce, 2013; Bukong et al., 2014). In addition to being involved in cancer progression, exosomal ncRNAs have been demonstrated to contribute to the onset of metabolic disorders, including osteoporosis, type 2 diabetes mellitus (T2DM) and diabetic nephropathy, and obesity (Li et al., 2021a; Mafi et al., 2021). NcRNAs are also involved in the pathogenesis and progression of neurodegenerative diseases, such as Parkinson’s and Alzheimer’s diseases (Jea et al., 2019; Dorostgou et al., 2022; Vakili et al., 2023). NcRNAs encapsulated in exosomes are even associated with infectious diseases (e.g., viral hepatitis) and autoimmune disorders, such as rheumatoid arthritis (RA) (Wang et al., 2018b; Jiao et al., 2021). The aforementioned pathological roles of exosomal ncRNAs can help researchers find the ambiguous aspects of disease pathophysiology and develop novel diagnostic and therapeutic targets to improve disease management (Dea et al., 2014; Lv et al., 2020a; Li et al., 2021a). Especially, in the case of cancer and related pathological mechanisms, drug resistance is of great significance, as it results in conditions where chemotherapy does not work. Regarding the global incidence of BC and the role of chemoresistance in its ineffective treatment, potential interactions between exosomal ncRNAs and BC drug resistance will be comprehensively discussed as follows.

## 6 Molecular mechanisms by which exosomal ncRNAs interfere with BC drug resistance

**FIGURE 1:**
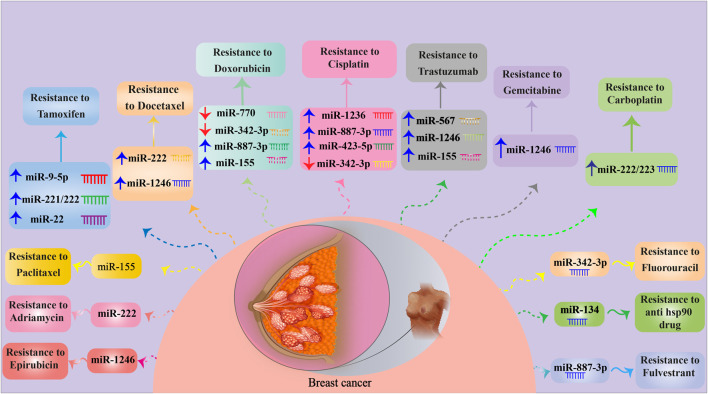
MiRNAs involved in BC drug resistance. For detailed information about signaling pathways targeted by miRNAs, see Table 1. ↑ and ↓ indicate up- and down-regulation, respectively.

**FIGURE 2:**
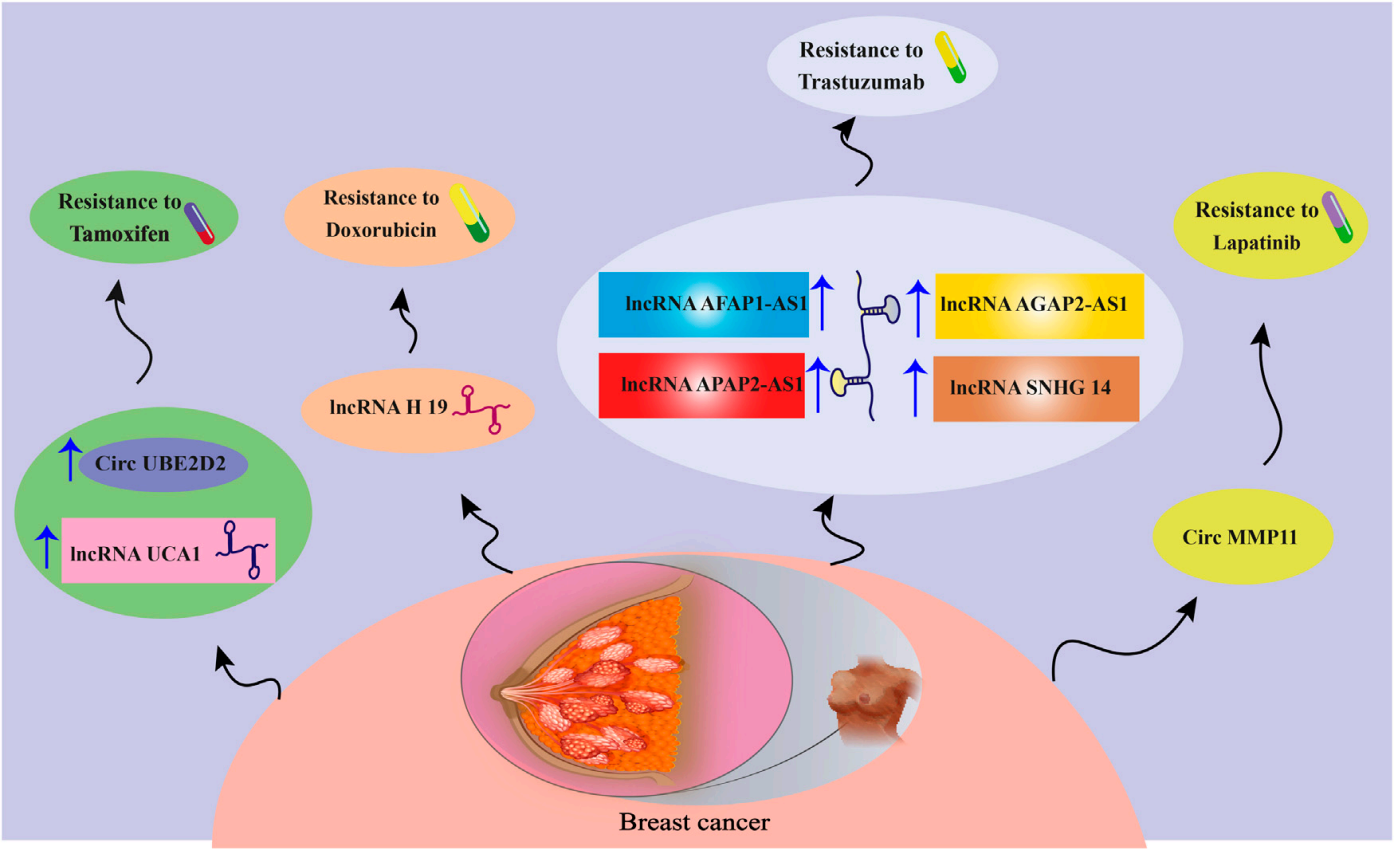
A schematic view of the effects of different lncRNAs and circRNAs on BC drug resistance. For more information about the relevant molecular mechanisms, visit Table 1.

Exosomes transport biologically active molecules between various cell types to mediate the initiation and progression of BC. In this regard, exosomal ncRNAs have a remarkable impact on a variety of tumor biology-related processes, including tumor growth, metastasis, migration, and drug resistance. Recently, it has been discovered that exosomal ncRNAs, particularly exosomal miRNAs, lncRNAs, and circRNAs, are involved in BC regulatory mechanisms (Chen et al., 2021; Singh et al., 2022). Exclusively, the latest findings on the crosstalk between exosomal ncRNAs (miRNAs, lncRNAs, and circRNAs) and BC drug resistance will be reviewed in the upcoming sections (Figures 1, 2).

### 6.1 Exosomal miRNAs and BC chemoresistance

MiRNAs are considered to be central to the regulation of genes linked to BC drug resistance (Fu and Tong, 2020; Purwanto et al., 2021; Liu et al., 2022). Several mechanisms modulated by miRNAs have been recognized in relation to BC chemoresistance; targeting genes involved in drug efflux and metabolism; cellular responses to chemotherapeutics (e.g., apoptosis, cell cycle arrest, and DNA repair); epigenetic alterations (e.g., DNA methylation and histone modifications); and deregulation of drug targets and receptors, which are the best examples in the field (Kutanzi et al., 2011). On the other hand, the miRNA-containing exosomes secreted by tumor cells can possibly be internalized by other cells to deliver gene modulatory characteristics to the recipient cells (Bach et al., 2017; Schwarzenbach, 2017; Salehi et al., 2022). As an example, it has been demonstrated that endothelial cell-derived exosomal miR-503, which is increased after neoadjuvant chemotherapy, can impair tumor growth, invasion, and proliferation in BC by inhibiting the expression of cyclin D2- and D3-encoding genes (CCND2 and CCND3) (Bovy et al., 2015). MiRNA expression profiles in BC cells were also reported to be either upregulated or downregulated in response to three chemotherapy drugs: docetaxel, epirubicin, and vinorelbine. In the same study, 12 of 22 upregulated miRNAs showed a significant upregulation, following pre-neoadjuvant chemotherapy, and thus play key roles in several pathways related to BC drug resistance, such as p53, Wnt, MAPK, and ErbB signaling pathways (Zhong et al., 2016). Here, the exosomal miRNA–drug resistance network in BC is highlighted with a focus on the chemotherapy drug subtypes.

#### 6.1.1 Tamoxifen

Tamoxifen (TAM) is an effective FDA-approved chemotherapeutic agent to combat ERα-positive breast tumors, particularly those in premenopausal patients. However, it has many adverse effects attributed to its estrogenic activities in other tissues (Cuzick and Baum, 1985; Freedman et al., 2003; Lumachi et al., 2013; Cuzick et al., 2015). TAM efficacy is attenuated by the development of drug resistance through a wide range of underlying mechanisms orchestrated by exosomal miRNAs (Ali et al., 2016; Yao et al., 2020). Liu *et al.* declared that exosomes derived from TAM‐resistant MCF-7 (ER-positive) cells could transfer miR-9-5p to TAM‐sensitive MCF-7 cells, resulting in the inhibition of cell apoptosis and promotion of MCF-7 resistance to TAM through downregulating the expression of the adiponectin gene (ADIPOQ) (Liu et al., 2021). Furthermore, the expression of miR-9-5p was up-modulated in BC tissue more than in normal breast tissue, and the increased miR-9-5p was also found to be associated with reduced ER expression in BC (Barbano et al., 2017). ADIPOQ, located on chromosome 3q27 and encoding adiponectin, can be linked to BC-related cell invasion (Mantzoros et al., 2004; Falk Libby et al., 2016). In addition, it has been elucidated that ADIPOQ overexpression induces autophagy and apoptosis in BC cells by activating the serine/threonine protein kinase 1/liver kinase B1 (STK11/LKB1)-associated AMP-activated protein kinase-Unc-51-like kinase 1 (AMPK–ULK1) pathway to decrease BC growth and progression (Chung et al., 2017; Zhang et al., 2017).

TAM-resistant MCF-7 cells can also induce TAM resistance in sensitive cells by propagating the exosomes containing miR-221/222 in ER-positive BC. In the presence of TAM, TAM-resistant MCF-7 cell-derived exosomes suppressed apoptosis and stimulated the colony-forming ability in TAM-sensitive MCF-7 cells. Exosomal miR-221/222 significantly inhibited the expression of P27 and ERα, promoting TAM resistance in recipient cells (Wei et al., 2014). Consistently, previous studies also demonstrated that miR-221/222 was notably upregulated in TAM-resistant MCF-7 cells and possibly involved in TAM resistance by regulating the expression of p27/Kip1 (Miller et al., 2008). In contrast, a recent evaluation indicated that miR-221/222 suppression could abolish TAM resistance, mediated by restoring the expression of ERα and PTEN (Ouyang et al., 2021).

According to the study conducted by Gao *et al.*, CD63^+^ cancer-associated fibroblasts (CAFs) secreted miR-22-enriched exosomes that could mitigate ERα expression and activate the PI3K/AKT pathway via PTEN downregulation to promote TAM resistance in BC cells (Gao et al., 2020). In addition, loss of ERα expression and CAFs is linked to a poor response of BC cells to TAM, with the suppression of the activity of CD63^+^ CAFs to enhance TAM sensitivity in BC cells, *in vivo* (Gao et al., 2020). CAFs originally constitute the major stromal components of the breast tumor microenvironment, indicating their effect on promoting cancer progression and mediating chemoresistance (Su et al., 2018; Ershaid et al., 2019). Activation of the PI3K/AKT signaling pathway can induce TAM resistance in BC cells, which may result in a decrease in ERα expression (Toska et al., 2017; Mills et al., 2018).

#### 6.1.2 Docetaxel

Although docetaxel (DOC)-based chemotherapy is an effective neoadjuvant approach to improve survival outcomes in BC patients (Heys et al., 2002), chemoresistance mediated by ncRNAs is almost unavoidable (Brown et al., 2004; Huang et al., 2018). According to the evaluations of Chen *et al.*, exosomes from DOC-resistant MCF-7 cells play substantial roles in transmitting DOC resistance to recipient DOC-sensitive cells through the delivery of responsible miRNAs. This study reported the top 20 most common miRNAs in DOC-resistant BC cell-derived exosomes involved in axon guidance, MAPK signaling pathway, cell cycle regulation, Wnt signaling, and TGF-β signaling cascade, which result in treatment failure when upregulated (Chen et al., 2014a). There are several exosomal miRNAs, being up- and down-modulated, secreted from adriamycin- and DOC-resistant BC cells. These BC cells can transfer miR-100, miR-30a, and miR-222 to drug-sensitive cells to modulate cell cycle distribution and drug-induced apoptosis. Exosomal miR-222 derived from DOC-resistant cells could also induce chemoresistance by decreasing PTEN expression in recipient cells (Chen et al., 2014b). In parenthesis, PTEN is an essential tumor suppressor and a pivotal component of the PI3K/PTEN/Akt signaling pathway associated with cellular processes (McCubrey et al., 2006; Steelman et al., 2008; Xia et al., 2020). PTEN underexpression was reported to serve as a predictive marker for poor outcomes in BC patients (Xu et al., 2017).

Further bioinformatics analyses found several overexpressed miRNAs, such as let-7a, let-7c, miR-103a, let-7b, miR-16, miR-23a, miR-27a, miR-23b, and miR-30a, as well as underexpressed miRNAs, such as miR-25, miR-130a, miR-20b, miR-425, miR-4725-5p, miR-455-3p, miR-551, and miR-92, in exosomes originated from DOC-resistant MCF-7 cells (Chen et al., 2019a). The overexpressed miRNAs mostly target the signaling pathways involved in the regulation of stem cell pluripotency and the TGF-β, FOXO, MAPK, and Wnt signaling pathways. However, the MAPK, TGF-β, FOXO, mTOR, and PI3K/Akt signaling pathways were found to be major targets for underexpressed miRNAs (Sebolt-Leopold and Herrera, 2004; Wang et al., 2008; Loh et al., 2013; Coomans de Brachène and Demoulin, 2016; Dey et al., 2017; Chen et al., 2019a; Movahedpour et al., 2022b). CCND1 and PTEN are considered the most common exosomal miRNA targets with high and low expression levels, respectively (Chen et al., 2019a). Overexpression of CCND1, the pivotal factor for cell transition from the G1 to the S phase, was reported in 50% of human BCs (Elsheikh et al., 2008). Another investigation also demonstrated that CCND1 organized the miRNA signature that induced the Wnt/β-catenin signaling pathway, serving as downstream and/or upstream targets of the Wnt/β-catenin axis (Wang et al., 2018c).

*Ccng2* was found to be downregulated in BC and served as a gene directly targeted by miR-1246. MiR-1246 has been reported to be overexpressed in human BC cells, particularly metastatic BC MDA-MB-231 cells (ER-negative). Exosomes derived from drug-resistant MDA-MB-231 cells can reduce apoptosis and promote the migration, invasion, and resistance to DOC, epirubicin (Liu et al., 2022), and gemcitabine (GEM) in non-malignant cells through transferring the aforementioned miRNA, i.e., miR-1246, leading to suppression of *Ccng2* gene expression (Sakha et al., 2016; Zhong et al., 2016; Li et al., 2017a). *Ccng2*, as a tumor suppressor gene, has a close relationship with cell cycle, DNA damage, and p53-related pathways (Bates et al., 1996; Montagner et al., 2012; Chang et al., 2015; Zimmermann et al., 2016). MiR-1246-mediated targeting of *Ccng2* also promotes cancer progression and chemoresistance in other cancers (Hasegawa et al., 2014; Lin et al., 2018).

#### 6.1.3 Doxorubicin

TNBCs are usually reported with a poorer prognosis, rapid progression, early metastasis, and containing no effective molecular targets for chemotherapy (Haffty et al., 2006). Doxorubicin, sold under the brand name adriamycin (ADM), is an anthracycline agent, which is considered an effective chemotherapeutic to treat BC (Jones et al., 1987; Wang et al., 2015). Nevertheless, doxorubicin resistance results in unsuccessful BC chemotherapy through different mechanisms, and overcoming the resistance can be a promising achievement in BC management (Nabholtz et al., 2003; Koike Folgueira et al., 2005; Lovitt et al., 2018). In this context, miR-770 was demonstrated to be overexpressed in chemo-sensitive TNBC cells, while it was downmodulated in chemoresistant cells. Moreover, overexpression of miR-770 inhibited tumor metastasis and doxorubicin resistance by induction of apoptosis *in vivo* and *in vitro*. MiR-770 is transferred by exosomes and causes chemosensitivity in cancer cells via the downregulation of stathmin 1 (STMN1) and suppressed cell invasion and migration by modification of the epithelial–mesenchymal transition (EMT) pathway (Li et al., 2018b). STMN1 or oncoprotein 18 is an essential cytoplasmic phosphoprotein responsible for cellular microtubule dynamics and depolymerization, involving cell cycle progression (Marklund et al., 1996; Rubin and Atweh, 2004). STMN1 participates in metastasis and chemoresistance of BC (Kuang et al., 2015; Kuang et al., 2016; Obayashi et al., 2017).

Santos et al. (2018) declared that exosomes separated from breast cancer stem cells (CSCs) and doxorubicin- and paclitaxel-resistant cells (i.e., MDA-MB-231 and MCF-7) could be transferred to recipient-sensitive cells, resulting in stimulation of migration and drug resistance through miR-155 delivery. Functionally, miR-155 upregulation was linked to the increased levels of B cell-specific Moloney murine leukemia virus integration site 1 (BMI1), snail family transcriptional repressor 1 (SNAI1 or SNAIL), SNAI2 (SLUG), enhancer of zeste homolog 2 (EZH2), and SRY-box transcription factor 9 (SOX9) and repression of E-cadherin in resistant cells, demonstrating molecular changes in EMT. MiR-155 also decreased the C/EBP-β, TGF-β, and FOXO-3a expression in sensitive cells co-cultured with exosomes from CSCs and chemoresistant cells (Santos et al., 2018). CSCs have a self-renewal ability, which is in line with tumor recurrence, resistance, and metastasis against chemotherapeutic agents (Lee et al., 2016). These cells can arise from epithelial cells subjected to EMT, determined by E-cadherin loss of expression accompanied by the upregulation of transcription factors such as BMI1 and EZH2. Therefore, the epithelial cell transformation into a mesenchymal state was triggered, resulting in the development of aggressive cancerous cells (Hiscox et al., 2006; Kajiyama et al., 2007; Dave et al., 2012; Proctor et al., 2013). Consistently, accumulating evidence has revealed the effects of overexpressed miR-155, as an oncomiR, on the development of chemoresistance in BC (Johansson et al., 2013; Ouyang et al., 2014; Shen et al., 2015; Yu et al., 2015; Chiu et al., 2016; Li et al., 2017b).

Sprouty2 was found to be deregulated in BC through involvement in metastasis- and invasion-related pathways targeted by miRNAs (Li et al., 2013). On the other hand, cyclin-dependent kinase (CDK) inhibitor p27, an essential factor in cancer cell cycle arrest, autophagy, and angiogenesis, as well as the PTEN, is targeted by miRNAs in regulating BC drug resistance (Li et al., 2013; Zhong et al., 2013). Further investigations have detected that exosomes secreted by ADM-resistant MCF-7 BC cells could transfer drug-resistance characteristics to recipient-sensitive cells through miR-222 delivery (Yu et al., 2016). Activation of the PTEN/Akt/FOXO1 signaling pathway is believed to be responsible for the underlying mechanisms through which miR-222 affects ADM resistance in BC cells. MiR-222 causes PTEN to be suppressed and also results in Akt overexpression and FOXO1 downmodulation. The suppression of the PTEN/Akt/FOXO1 axis is linked to increased ADM sensitivity in BC cells (Shen et al., 2017). The upregulation of FOXO, following PTEN activation and Akt suppression, has represented tumor-suppressive effects such as the induction of cell cycle arrest and/or cancer cell apoptosis (Greer and Brunet, 2005). More recently, a study exhibited that lncRNA-GAS5 could alleviate ABCB1-mediated ADM resistance of BC cells by repressing miR-221-3p, which directly targeted Dickopf Wnt signaling pathway inhibitor 2 (DKK2). Subsequently, the Wnt/β-catenin signaling axis was activated, leading to the inhibition of ABCB1 expression and further alleviation of ADM resistance (Chen et al., 2020a).

#### 6.1.4 Anti-Hsp90 drugs

Anti-Hsp90 drugs, such as 17-AAG or PU-H71, have shown promising anticancer effects in TNBC therapy during pre-clinical investigations (Eiseman et al., 2005; Caldas-Lopes et al., 2009; Proia et al., 2014). O’Brien *et al.* observed that EV-derived miR-134 was downmodulated in Hs578T TNBC cells compared to normal breast cells, in which it was shown to be underexpressed in TNBC aggressive clonal variant (Hs578 Ts(i)_8_) cells. Indeed, miR-134 loss is linked to elevated cellular aggressiveness. Also, miR-134 could act as a potential tumor suppressor by inhibiting STAT5B, which, in turn, reduced the Hsp90 and Bcl-2 expression levels. It has previously been revealed that STAT5B is involved in BC tumorigenesis and increases the transcription of Hsp90 (Perotti et al., 2008), which enhances the survival and apoptotic resistance of BC cells (Workman et al., 2007; Gallerne et al., 2013). Direct delivery of miR-134 caused suppression of TNBC cell proliferation and promotion of cisplatin-induced apoptosis, whereas delivery of exosomal miR-134 inhibited cell proliferation, migration, and invasion, as well as the enhancement of sensitivity to anti-Hsp90 drugs (O'Brien et al., 2015). MiR-134 originates from the 14q32 locus, a region usually deleted in tumor progression (O'Brien et al., 2015; Takayama et al., 1992; Kerangueven et al., 1997; Bando et al., 1999; Haller et al., 2010; Gattolliat et al., 2011). In line with this finding, other observations have reported that miR-134 has a tumor-suppressive role and its levels are inversely associated with tumor progression (Li et al., 2012; Sarver et al., 2013; Yin et al., 2013).

#### 6.1.5 Cisplatin

Cisplatin (DDP) is another effective chemotherapeutic agent for BC therapy, especially for TNBCs that show an ineffective response to anti-HER-2 therapies (Silver et al., 2010). Although patients initially exhibit positive responses against DDP, drug resistance remains a major challenge that causes failure in treatment (Smith et al., 2007). Jia *et al.* have revealed that adipose mesenchymal stem cell (ADMSC)-derived exosomes, containing miR-1236, can decrease BC cell resistance against DDP by inhibiting the solute carrier family 9 member A1 (SLC9A1) Na^+^/H^+^ anti-porter and inactivating the Wnt/β-catenin signaling pathway (Jia et al., 2020). Indeed, miR-1236 directly binds to SLC9A1 and suppresses its expression, which is notably overexpressed in DDP-resistant BC cells. In addition, ADMSC-derived exosomes induce a further increase in caspase-3, promoting cell apoptosis in DDP-resistant BC cells (Jia et al., 2020; Asadi et al., 2022). Effective functions of ADMSC-derived exosomes in cancer control and treatment have been reported to be exerted by regulating various cellular behaviors, like proliferation, migration, and apoptosis, by means of miRNA cargos (Reza et al., 2016; Hong et al., 2019). It has been noted that SCL9A1 acts as an oncogene and is involved in the development of drug-resistant BC cells (Chen et al., 2019b). SLC9A1 was also discovered to be a Wnt/β-catenin signaling activator, participating in BC carcinogenesis, metastasis, and tumor progression (Prosperi and Goss, 2010; Sun et al., 2018b). Recently, it has been revealed that about 60 miRNAs, particularly miR-423-5p, miR-370-3p, and miR-373, are significantly upregulated in exosomes from DDP-resistant TNBC cells compared to DDP-sensitive ones. DDP-resistant cell-derived exosomes could induce DDP resistance in recipient cells by miR-423-5p delivery, which leads to P-gp overexpression, invasion, and migration, as well as apoptosis suppression (Wang et al., 2019a).

Yu *et al.* indicated the underexpression of miR-342-3p in samples collected from patients with metastatic and refractory BC types. Their investigations further revealed that the MSC-derived exosomes consisting of miR-342-3p could inhibit invasion, metastasis, and chemoresistance of BC cells to doxorubicin, fluorouracil, and cisplatin by suppressing the inhibitor of differentiation 4 (ID4). Also, ID4 silencing significantly reduced tumor growth and drug resistance and influenced the EMT by upregulating the E-cadherin and downregulating the N-cadherin and SNAIL (Yu et al., 2022). A similar investigation on TNBC patients declared that miR-342-3p loss of function contributed to metabolic carcinogenic pathways in TNBC by overexpression of MCT1 (Romero-Cordoba et al., 2018).

Recent *in vivo* and *in vitro* findings have shown that exosomes derived from MDA-MB-231 cells developed resistance-related mechanisms in BC cells by transferring miR-887-3p, which was overexpressed in BC cells. MiR-887-3p induced drug resistance through negative BTBD7 regulation, resulting in the activation of the Notch1/Hes1 signaling pathway (Wang et al., 2022a). In contrast, when miR-887-3p is inhibited in exosomes, drug resistance and tumor development are reduced, while BC cell apoptosis is increased (Wang et al., 2022a). Upregulation of miR-887-3p was observed in BC cell lines, and the suppressed miR-887-3p improved BC cell sensitivity to 5-fluorouracil therapy (Lv et al., 2020b). BTBD7 plays an important role in tumorigenesis, in which its expression can be declined in BC cells and tissues. BTBD7 expression is associated with low recurrence and repressed BC progression through inactivating Notch1 signaling (Chen et al., 2020b). Notch1 participates in cell growth, differentiation, and apoptosis. Notch1 activation was also proven in TNBC, as a facilitator of TNBC formation (Miao et al., 2020).

#### 6.1.6 Trastuzumab

Currently, trastuzumab (TZB), an anti-HER2 monoclonal antibody, has been introduced as an effective agent to cure human HER-2-positive BC (Daniels et al., 2018), while its effectiveness can mostly be restricted by chemoresistance-associated mechanisms (Adamczyk et al., 2018). In this context, Han *et al.* showed significant downregulation of miR-567 in TZB-resistant BC cells compared to the sensitive ones. Overexpression of miR-567 reversed TZB resistance, but miR-567 downregulation could induce TZB resistance, *in vivo* and *in vitro*. Functionally, miR-567 can be transferred to recipient BC cells by packaging into exosomes and reverses the chemoresistance by suppressing the autophagic flux, which is mediated by inhibiting the expression of *Atg5*, post-transcriptionally (Han et al., 2020). Autophagy-related gene 5 (*Atg5*) has a key function in the early stages of autophagy, i.e., autophagosome formation, and is associated with cell differentiation and carcinogenesis (Le Bars et al., 2014; Rai et al., 2019). Growing evidence has demonstrated the essential role of autophagy in preserving the survival of tumor cells in adverse conditions (Degenhardt et al., 2006). Additionally, the inhibitory functions of miR-567 in the oncogenesis of BC have been proven (Bertoli et al., 2017). Another investigation elucidated that exosomal miR-155 and miR-1246 were the principal overexpressed miRNAs in HER2-positive BC with TZB resistance, suggesting a remarkable prognostic value for these miRNAs (Zhang et al., 2020).

### 6.2 Exosomal lncRNAs and BC chemoresistance

#### 6.2.1 Tamoxifen

The overexpression of exosomal lncRNA urothelial carcinoma-associated 1 (UCA1), isolated from TAM-resistant LCC2 cells compared to TAM-sensitive MCF-7 cells, was an interesting finding in the field of exosomal lncRNA–BC drug resistance relationships. Exosome-transmitted lncRNA UCA1 was linked to TAM resistance in ER-positive BC cells, which might act by suppressing cleaved caspase-3 expression and cell apoptosis (Xu et al., 2016). The oncogenic function of UCA1 in BCs was identified through recruiting various mechanisms, such as inhibition of p27 (Huang et al., 2014) and acting as a miR-143 sponge (Tuo et al., 2015).

Chen *et al.* reported an association between the expression of myeloid-specific lncRNA and hypoxia-inducible factor 1α (HIF-1α)-stabilizing lncRNA (HISLA) in tumor-associated macrophages (TAMs) with poor chemotherapeutic responses and survival rates in BC patients (Chen et al., 2019c). The findings revealed that EV-mediated transmission of HISLA promoted apoptotic resistance and aerobic glycolysis in BC cells. Functionally, HISLA suppressed the hydroxylation and degradation of HIF-1α by blocking the interaction between HIF-1α and prolyl hydroxylase domain protein 2 (PHD2) (Chen et al., 2019c). Metabolic reprogramming is an indicator for developing cancer cells (Hay, 2016), with frequently reported deterioration of glucose metabolism in BC (Lloyd et al., 2016; Ibrahim-Hashim et al., 2017). HIF-1α, an oxygen-sensing transcription factor, is known to determine glucose metabolism by oxidation or glycolysis in cancer cells, which could be considered adaptive changes to TAM circumstances (Hsu and Sabatini, 2008; DeBerardinis and Chandel, 2016). Under physiological conditions, HIF-1α can be quickly hydroxylated and degraded by PHD2; however, multiple factors involved in TAM may modulate PHD2 activity to inhibit HIF-1α degradation, leading to the maintenance of HIF-1α protein, and promote aerobic glycolysis in cancer cells (Hsu and Sabatini, 2008; Semenza, 2012; Semenza, 2013).

#### 6.2.2 Trastuzumab

LncRNA SNHG14 is a well-studied lncRNA promoted in TZB-resistant BC cells through overexpression of the poly (a)-binding protein cytoplasmic 1 (PABPC1) gene, leading to Nrf2 signaling activation (Dong et al., 2018). PABPs can participate in sequence-specific interactions with single-stranded poly (A) via an RNA recognition motif (RRM). In the cytoplasm, PABPs are categorized as PABPC, and inside the nucleus, they are known as PABPN1 (Burgess et al., 2011).

The lncRNA AGAP2 antisense RNA 1 (AGAP2-AS1) is another lncRNA having high expression levels in TZB-resistant BC cells and promotes resistance by reducing TZB-induced apoptosis. Thus, silencing of AGAP2-AS1 could re-sensitize BC cells to TZB-induced cytotoxicity. AGAP2-AS1 could also disseminate TZB resistance through packaging into exosomes (Zheng et al., 2019). According to the study conducted by Qian *et al.*, exosomal AGAP2-AS1 caused TZB resistance by inducing autophagy in HER2-positive BC cells. In detail, the corresponding exosomal lncRNA exerts its effects by promoting *Atg10* expression. Mechanistically, the embryonic lethal abnormal visual system (ELAV)-like RNA-binding protein 1 (ELAVL1) interacted with and stabilized the AGAP2-AS1, and subsequently, the AGAP2-AS1–ELAVL1 complex directly attached to and promoted H3K4 trimethylation, as well as H3K27 acetylation at the *Atg10’s* promoter region, thus resulting in activation of *Atg10* transcription (Qian et al., 2021). AGAP2-AS1-targeting antisense oligonucleotides (ASOs) promoted TZB-related cytotoxicity. The elevated serum levels of exosomal AGAP2-AS1 were linked to poor response against TZB treatment (Luo et al., 2020; Qian et al., 2021).

#### 6.2.3 Doxorubicin

It has been found that lncRNA H19 is upregulated in doxorubicin-resistant BC cells, while its suppression significantly decreases doxorubicin resistance by reducing cell viability, colony‐forming ability, and inducing apoptosis. H19 can disseminate doxorubicin resistance among sensitive cells by being packaged into exosomes (Wang et al., 2020). H19 levels have been reported to be elevated in serum samples from patients unresponsive to doxorubicin (Wang et al., 2020). H19 also participates in the onset and development of BC through multiple mechanisms (Collette et al., 2017). For instance, H19 results in the overexpression of transcriptional factor LIN28, which has a critical role in BC stem cell maintenance, by sponging miRNA let-7 (Peng et al., 2017). Moreover, a previous study has demonstrated that H19 suppresses apoptosis in ERα‐positive BCs, thereby promoting paclitaxel (PTX) resistance by repressing transcription of the *Bik* pro-apoptotic gene (Si et al., 2016). H19 was also reported to mediate doxorubicin resistance in BC cells by regulating the cullin4A–MDR1 signaling pathway (Zhu et al., 2017).

### 6.3 Exosomal circRNAs and BC drug resistance

#### 6.3.1 Tamoxifen

The upregulation of circRNA UBE2D2 (circ-UBE2D2) was elucidated by Hu *et al.* in TAM-resistant ERα-positive BC cells. TAM-resistant cells could release exosomes containing circ-UBE2D2, thus inducing TAM resistance in drug-sensitive BC cells. Mechanistically, circ-UBE2D2 interacts with miR-200a-3p, and subsequently decreases its function, thereby promoting metastasis, cancer cell viability, and TAM resistance (Hu et al., 2020b). It has been shown that miR-200a-3p is a target for LINC00894-002 to contribute to TAM resistance in MCF-7 BC cells (Zhang et al., 2018). Previously, circ-UBE2D2 was displayed to be overexpressed in BC; therefore, blocking the circ-UBE2D2 could lead to the inhibition of BC tumorigenesis through targeting miR-1236 or miR-1287 (Wang et al., 2019b).

#### 6.3.2 Lapatinib

Despite lapatinib (LAP) being a small-molecule tyrosine kinase inhibitor with high levels of effectiveness and limited side effects for treating HER2-positive BC (Rusnak et al., 2001; Chan, 2006), innate or acquired LAP resistance has led to an obstacle for BC therapy (Liu et al., 2009; Wang et al., 2011). A recent assessment conducted by Wu *et al.* demonstrated the circ-MMP11 overexpression in LAP-resistant cells, which could be transported to sensitive cells through exosomes to induce cell viability, invasion, migration, and repressing apoptosis in LAP-sensitive cells. Regarding the underlying mechanism, circ-MMP11 serves as a potential sponge for miR-153-3p, increasing the expression of anillin (ANLN) (Wu et al., 2021). ANLN is upregulated in BC, whose up-regulation contributes to drug resistance in BC cells against doxorubicin (Zhou et al., 2015). Additionally, circ-MMP11 (hsa_circ_0062558) has been reported with carcinogenic effects by serving as a miR-1204 competitive endogenous RNA (ceRNA) (Li et al., 2020b). MiR-153-3p is another circRNA with critical inhibitory roles in BC progression and LAP resistance (Yu et al., 2018).

#### 6.3.3 Non-exosomal circRNAs

Although limited evidence is available with respect to the effects of exosomal circRNAs on the development of BC drug resistance, a large number of investigations have reported the crucial functions of non-exosomal circRNAs in chemotherapy resistance during BC progression. For instance, Wang *et al.* demonstrated that circATXN7 could act as a miR-149-5p sponge to upregulate homeobox A11 (HOXA11), resulting in increased doxorubicin resistance in BC cells (Wang et al., 2022b). Moreover, Hsa_circ_0092276 promoted resistance to doxorubicin in BC cells by modulating the ATG7 via sponging miR-384, leading to activated autophagy (Wang et al., 2021a).

CircWAC is another cirRNA that can induce PTX resistance in TNBC cells by suppressing the miR-142, a tumor suppressor miRNA, inducing the overexpression of WWP1 and activation of the PI3K/Akt pathway (Wang et al., 2021b). Consistently, circ_0006528 was found to partially participate in PTX resistance in BC cells by stimulating the expression of CDK8 by sponging miR-1299 (Liu et al., 2020). Further studies showed that the upmodulated circ-RNF111 was associated with PTX resistance, as well as cancer cell viability, cell invasion, and glycolysis. Indeed, circ-RNF111 is negatively correlated with miR-140-5p expression, hence triggering E2F3 in BC tissues (Zang et al., 2020).

Recently, it has been shown that circ_0085495 causes ADM resistance in BC cells by triggering the inhibition of miR-873-5p, and consequently the overexpression of integrin ß1 (Xie and Zheng, 2022). Similarly, the role of circ_0001667 in developing ADM resistance in BC cells was observed in another study, in which circ_0001667 knockdown attenuated ADM resistance, decreased cancer cell proliferation, and enhanced apoptosis by depleting nuclear receptor co-activator 3 (NCOA3) via releasing miR-4453 (Cui et al., 2022). Zhu *et al.* revealed that circFBXL5 might enhance 5-FU resistance in BC cells by promoting cell migration and invasion and inhibiting apoptosis by regulating the miR-216b/HMGA2 axis (Zhu et al., 2021). Another investigation identified that circ_0001598 promoted TZB resistance by modulating the miR-1184/programmed death-ligand 1 (PD-L1) signaling pathway in HER2-positive BC (Huang et al., 2021). CircFAT1 also promoted oxaliplatin resistance in BC through the activation of Notch and Wnt signaling pathways, principally through regulation of the miR-525-5p/SKA1 axis (Yao et al., 2021). CircUBAP2, another circRNA involved in drug resistance, could increase DDP resistance in TNBC cells by serving as a ceRNA for miR-300 to overexpress the anti-silencing function 1B histone chaperone (ASF1B), resulting in activation of the PI3K/Akt/mTOR signaling pathway (Wang et al., 2022c).

On the other hand, several circRNAs have shown a tumor-suppressive function; in this regard, hsa_circ_0025202 could reduce cancer cell proliferation, hinder tumor growth, promote apoptosis, and enhance TAM sensitivity in BC cells. Also, it has been shown that hsa_circ_0025202 exerts its functions through binding to miR-197-3p, thereby increasing HIPK3 expression (Li et al., 2021b). Furthermore, circ_0025202 was found to improve TAM sensitivity in BC cells by targeting miR-182-5p, leading to the regulation of FOXO-3a expression and activity (Sang et al., 2019).

## 7 Conclusion and perspectives

**FIGURE 3:**
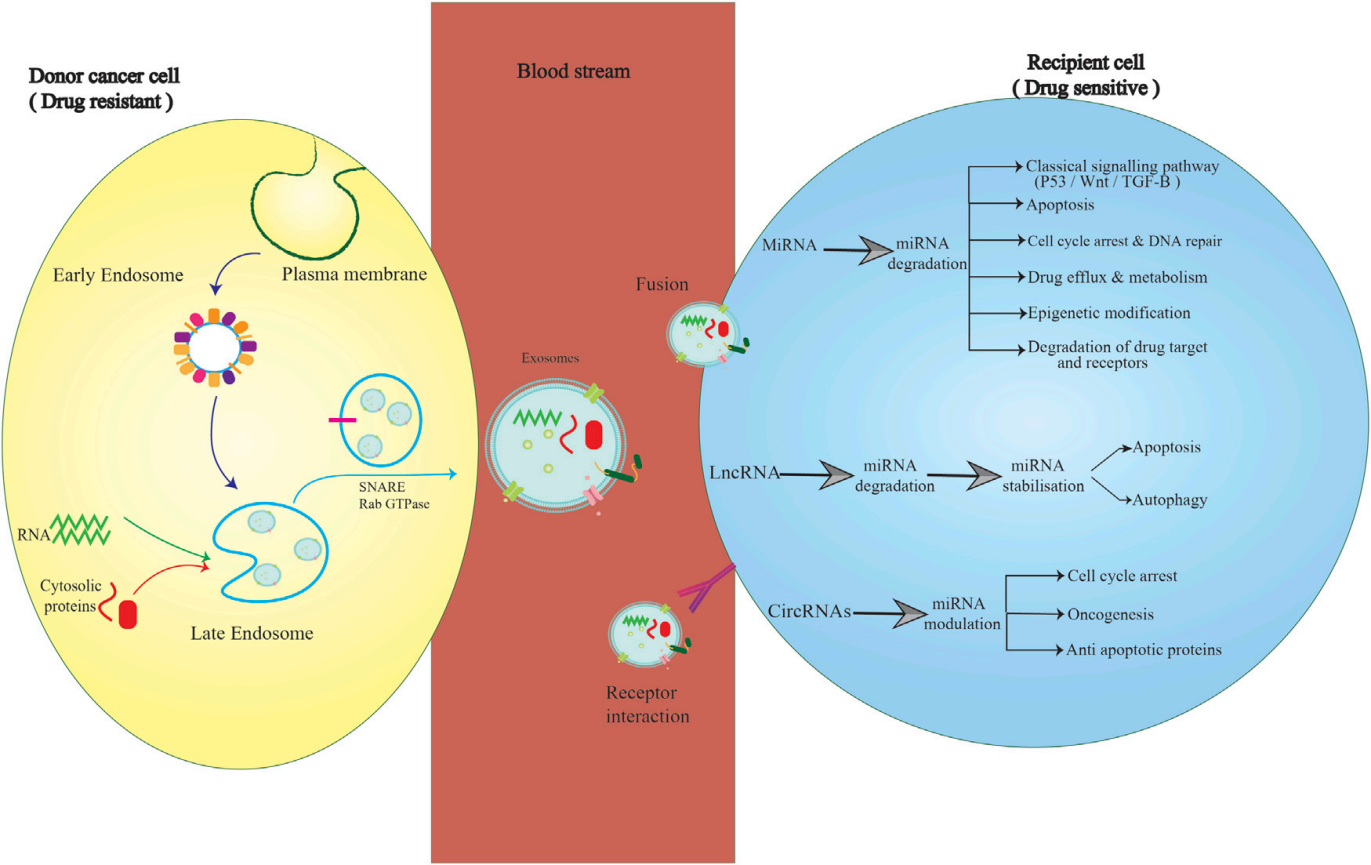
Exosomal delivery of ncRNAs between cancer and non-cancer cells. Exosomes derived from drug-resistant BC cells encapsulate and then deliver miRNAs, lncRNAs, and circRNAs to drug-sensitive cells, thus promoting multiple signaling pathways in recipient cells. This characteristic can be considered the basis for designing more effective chemotherapeutic approaches by attenuating drug resistance.

Chemoresistance, whether inherent or acquired, contributes to poor prognosis and treatment failure in cancer patients. Elucidating the underlying mechanisms of drug resistance development might help researchers to suggest a more effective approach for treating patients. Considering drug resistance, BC therapy has proven to be complicated. Recently, exosomal ncRNAs have been surprisingly found to regulate drug resistance in BC cells and TAM. Beyond drug resistance, exosomal ncRNAs play a substantial role in the development of cancer-related behaviors, such as cell proliferation, angiogenesis, and migration, by regulating several molecular mechanisms (Figure 3). As exosomal ncRNAs can be transferred through the bloodstream, they seem to have tremendous potential as diagnostic markers in clinical settings. Moreover, loading ncRNAs, as well as ncRNA agonists or inhibitors, into particular exosomes, and then delivering the exosomes into the bloodstream to be distributed among cancer cells can provide an exosome-based approach to treat malignancies. However, a wide variety of molecular mechanisms directing exosomal ncRNAs to a certain human cell or organ have remained unclear. In the current study, the contribution of exosomal ncRNAs and their target mechanisms/signaling pathways to BC drug resistance was highlighted in detail. The majority of findings reviewed here were the outcomes of *in vitro* evaluations, as there are limited human studies in this field. Notwithstanding, new insights were provided here that might help cancer researchers overcome drug resistance by designing exosomal ncRNAs-based therapeutics. Further assessments are definitely needed to elucidate the unclear aspects of BC chemoresistance in association with exosomal ncRNAs.
